# Similarities and Differences between Nurses' and Physicians' Clinical Leadership Behaviours: A Quantitative Cross-Sectional Study

**DOI:** 10.1155/2023/8838375

**Published:** 2023-12-13

**Authors:** Anoek Braam, Martina Buljac-Samardžić, Carina Hilders, Jeroen van Wijngaarden

**Affiliations:** ^1^Department Health Services Management & Organisation (HSMO) of the Erasmus School of Health Policy and Management, Erasmus University Rotterdam, Rotterdam, Netherlands; ^2^Reinier de Graaf Gasthuis, Delft, Netherlands

## Abstract

**Background:**

Being a nurse or physician in today's complex healthcare practice involves more than just responsibility for one aspect of care during one episode in a patient's care trajectory. Both professionals are expected to take on a clinical leadership role and contribute positively to the reduction of care fragmentation and help in spanning professional boundaries. Although nurses may be well placed to identify the needs for integration, they may lack the position and status (compared to physicians) to address those needs as leaders. The aim of this study is to analyse similarities and differences between nurses and physicians in clinical leadership roles within a hospital context and explore how this relates to their interdisciplinary collaborative behaviours and perception on their job.

**Method:**

A cross-sectional survey among physicians and nurses was conducted to measure clinical leadership, job satisfaction, workload, and interdisciplinary collaborative behaviours.

**Results:**

Our results suggest that nurses (*n* = 329) and physicians (*n* = 100) show similar clinical leadership behaviours, based on equivalent scores on the clinical leadership scale. However, physicians score higher on the global leadership scale indicating they are more likely to perceive themselves as leaders than nurses. As clinical leaders, both nurses and physicians are more likely to express interdisciplinary collaborative behaviours. Furthermore, physicians who scored higher on the clinical leadership scale reported higher satisfaction with their job, whereas, for nurses, their score on the clinical leadership scale did not relate to their job satisfaction.

**Conclusion:**

As nurses in hospitals have the most frequent and direct involvement with patients, it seems inevitable for them to act as clinical leaders to promote patient-centred care. However, nurses less often perceived themselves as clinical leaders while showing suitable behaviours. Future studies should focus on the strategies nurses use to exert their clinical leadership, and for example, if nurses require the use of more dominant strategies to effect change.

## 1. Introduction

Being a healthcare professional in today's complex healthcare practice involves more than just being responsible for *one* aspect of care during *one* episode in a patient's care trajectory [1, 2]. Professionals are expected to take a more holistic perspective and to be part of an integrated approach. Clinical leaders are seen as the “front-runners” in healthcare and regarded as being imperative to increase the integration of care [3–5]. Clinical leaders are expected to contribute positively by reducing care fragmentation and ensuring the spanning of professional boundaries [3]. According to Stanley and Stanley [4], there is a consensus in the literature that the role of a clinical leader can be fulfilled by every healthcare professional involved in direct clinical care. Literature suggests that the fundamental attributes that identify a good clinical leader and role models are as follows: being a supportive, approachable, and effective communicator; being a motivator and mentor for others, while remaining visible in clinical practice and having values and beliefs on excellence and quality [4, 5].

Based on the reviews on clinical leadership [4] and medical leadership [6] and the literature-defined core attributes [4, 5, 7–9], we argue that a clinical leader is a healthcare professional who is directly involved in clinical care and continuously puts effort in the improvement of care and inspires and motivates others to do the same. Clinical leadership is often regarded as an informal role that can be performed without this being delivered from a formal leadership position. Our definition uses the term healthcare professional purposely, as the clinical leadership role can and should be performed by physicians, as well as nurses. They are both expected to implement the changes necessary to meet current healthcare demands and to build bridges between domains.

Physicians and nurses in day-to-day healthcare practice clearly have different roles, reflecting their scope of practice and position towards patients [10]. The general public and many health professionals have for a long time perceived physicians as the leaders in patient care, while nurses were seen as playing a subordinate role [11, 12]. Therefore, it is not surprising that leadership involving physicians and nurses form two distinct themes in the literature. Studies on the formal leadership of physicians often focus on balancing or bridging the gap between management and medicine, especially as there is an increasing drive to see physicians take on significant leadership roles within the healthcare system [6, 11, 13]. Some studies also discuss the informal leadership role that physicians play in patient care. This is often referred to as medical leadership or medical management [8, 11]. Despite positive effects and growing attention in the literature to study nurse clinical leadership, informal nurse leaders are often seen as “rebels” [14]. Also within nursing, leadership is still equated with a formal leadership position [12, 14], while research advocates clinical leadership by nurses as an essential element for innovation and change towards integrated healthcare [15]. In particular, the potential impact of nurses is great because of their ability to identify areas for improvement at patient and organizational levels [9, 16]. Despite the differences of current leadership positions held by physicians and nurses, as clinical leaders, they are both expected to innovate healthcare, bridge domains to craft the practice of policy, play a role in implementing the changes necessary to meet current healthcare demands and, thus, fulfil a similar role [12, 17].

Although clinical leadership can be delivered by nurses and physicians and might even be a vital part of both of their day-to-day practice, there are differences that should be recognized. For example, physicians and nurses do not have the same leadership experience. They will have different educational backgrounds and different scopes of daily practice and hold different positions towards patients. So, understanding how both act as clinical leaders is central to understanding how clinical leaders can make changes to improve care [4]. However, to date, the authors are aware of only a few studies that address the similarities and differences between physicians' and nurses' clinical leadership and how their respective clinical leadership relates to patient, staff, and organizational outcomes [3, 18, 19]. Therefore, the aim of this study is to describe similarities and differences between clinical leadership behaviours of nurses and physicians within a hospital context and explore how clinical leadership behaviours relate to interdisciplinary collaborative behaviours, using a quantitative approach.

## 2. Background

Our first hypothesis relates to the notion that taking on the clinical leadership role may seem less natural for nurses. This is because physicians have more experience as informal leaders and are more likely to exert influence [20, 21]. In the traditional hierarchy, nurses often have a more subordinate role that discourages them to question or deviate from rules and regulations or seek a leadership role, even if the purpose is to benefit the patient [14, 21, 22]. Furthermore, research shows that nurses believe they lack the necessary knowledge and skills to perform a clinical leadership role [22].

  Hypothesis I: nurses are less inclined to assume a clinical leadership role than physicians.

Clinical leaders are regarded as imperative for integration of care, but it is unclear whether taking up this role is facilitated and supported enough, especially when it relates to nurses [23]. Research seems to suggest that nurses are intrinsically motivated to take on a clinical leadership role [24], which could also increase their job satisfaction. However, lack of time, lack of financial incentives, and lack of support from other health professionals discourage nurses taking on a clinical leadership role [25]. Physicians may even resist nurses taking on this role if it questions their (traditional) leadership position or creates unclear role boundaries [26–28]. If nurses have to fight resistance to take on this new role and are not financially (or any other way) rewarded, it may increase their workload and reduce their work satisfaction. For physicians, this may be different, as acting as an informal leader is already part of their role (albeit in a different context) and is embedded in their identity [8, 29]. We therefore expect physicians to be naturally inclined to take on a leadership role and not to perceive it as an increase in workload. As professional development is a strong predictor of physicians' job satisfaction [30], we expect that physicians who engage in clinical leadership roles will be more satisfied with their jobs.

  Hypothesis IIa: nurses' clinical leadership behaviours will lead to higher perceived workload but not necessarily higher job satisfaction.
  Hypothesis IIb: physicians' clinical leadership behaviours will not necessarily lead to higher perceived workload but will lead to higher job satisfaction.

Hospitals with physicians in management positions have been shown to deliver better quality and, overall, more effective services than hospitals with those with less clinician involvement [31]. This has been related to the ability of these physicians to bridge the gap between management and physicians. Some authors have suggested that physicians also should take the lead in breaking down medical silos [32, 33]. As clinical leaders who focus on balancing diverging perspectives and crossing specialist boundaries [34, 35], physicians should be able to improve relationships with physicians from other specialties.

  Hypothesis III: physician clinical leaders will act as bridge builders (towards physicians from other specialties).

Their roles in improving care mean it is inevitable that physician and nurse clinical leaders will cross paths. Their unique characteristics and professional expertise can be, on the one hand, complementary and, on the other hand, challenging [36]—complementary, as they both have their own scope of practice and values [36, 37], and challenging as they differ in beliefs about possible solutions and perceived barriers [36, 37]. Nurses perceive the hierarchy of professions as a barrier to their leadership development and their influence, if their voice is not recognized [36, 38]. From research on formal leadership physician-nurse dyads and interprofessional collaboration, we learn that explicit goals, understanding of the other profession, and respect for one another are important for a complementary physician-nurse relationship to actually work well [39, 40]. The literature shows that physicians and nurses are both open to and value interprofessional collaboration [36, 41, 42]. However, as described by other researchers, those physicians with more power are less likely to desire a collaborative relationship [42]. As such, we argue that, for a collaborative relationship between nurse and physician clinical leaders to occur, nurses need to show how they add value. Nurse clinical leader need to make physicians aware that, together, they can achieve more as a result of the synergy. Nurses might encounter resistance from physicians by trying to be acknowledged in their leadership role as physicians might feel threatened in their leadership position. This might lead to tension between nurses and physicians.

  Hypothesis IV: when nurses take on a clinical leadership role, this will negatively impact the relationship with physicians.

## 3. Methods

From October to December 2020, we conducted a cross-sectional survey among physicians and nurses in a Dutch hospital. This 481-bed-counting hospital is in addition to providing good basic care focused on training, science, and innovation, which results in several domains in which this hospital delivers demonstrably distinctive care compared to care provided in other hospitals. Furthermore, according to the organizations information, the position of nurses is highly valued, witnessed by a Nursing Leadership program. Nurses and physicians participated in a survey with overlapping and profession-specific questions.

### 3.1. Sampling

Convenience sampling was used to recruit the study participants. All physicians (*n* = 392) and nurses (*n* = 850) working in the hospital were considered eligible for participation in the study and received a direct link to the survey via email. The minimum sample size needed was 89 (per group) to reach a sufficient power (95%), effect size (0.15), and alpha (0.05), based on G-power version 3.1.9.7. The survey was built in Castor EDC, a highly secured, cloud-based electronic data capture platform [43]. Beforehand, three physicians and two nurses assessed their respective surveys to identify ambiguities and provide feedback. After the first email invitation, in total, six reminders were sent, with an interval of between one and two-and-a-half week(s), to health professionals who had not completed the survey. Due to the low response rate after the third reminder, we decided to hand out paper copies of the surveys. The paper copies were distributed by wards' head nurses. To return the survey, a sealable envelope was attached and the sealed envelope could be returned in an anonymous box at the ward. A sixth reminder via email was sent just before the Christmas holidays of 2020, where a raffle of 50 bottles of Champagne for everyone completing the survey before the start of 2021 was announced.

The questionnaire elicited respondents' background characteristics, such as gender, whether they held a formal leadership position, and tenure characteristics such as function and work experience (ranging from 1, <1 year, to 6, >21 years). As respondents might feel that responding to these questions reveals their identity, an opt out option was included to avoid dropouts. Respondents who did not answer all fifteen items of the instrument of main interest (clinical leadership) were excluded.

### 3.2. Measurements

#### 3.2.1. Leadership

We assessed clinical leadership of physicians and nurses using a translated version (to Dutch) of the Clinical Leadership Survey (CLS) [44]. We used the CLS for the following reasons: (i) the content of the questionnaire covered our definition of clinical leadership; (ii) it is a self-administered questionnaire measuring one's own leadership behaviour, which we considered suitable for use among health professionals that hold an informal leadership; (iii) the length of the questionnaire (15 items) is pragmatic for use among professionals with limited time; and (iv) although designed for nurses, it is still well-suited (with limited changes) to be administered with physicians.

The questionnaire is derived from Kouzes and Posner's model (1995) on transformational leadership and was adapted to reflect current clinical leadership practices. The text was back-translated (by a native English speaker) and then synthesised and reviewed by the target groups. The CLS assesses self-perceived transformational leadership behaviours based on 15 items. Participants are asked to assign the most appropriate rating on a five-point Likert scale (1 = almost never, 2 = occasionally, 3 = some of the time, 4 = most of the time, and 5 = almost always). The scale was reported to have Cronbach's alpha of 0.86 [44]. Our translated Dutch version of the CLS provided an acceptable Cronbach's alpha of 0.79 (for the whole sample of physicians and nurses), with a negligible difference between Cronbach's alpha for physicians 0.80 and nurses 0.79. The total clinical leadership score was an average from the 15 items and ranged from 1 to 5, with higher scores indicating more self-reported leadership behaviour.

Next to the CLS reflecting transformational leadership behaviours, a two-item global leadership scale was used. This scale was added to check to what extent respondents perceived themselves as leaders in their clinical practice. The global leadership asks respondents to rate the following: (a) the extent to which they perceived themselves as leaders and (b) the extent to which they demonstrated leader behaviour in their clinical practice on a five-point Likert scale (1 = almost never, 2 = occasionally, 3 = some of the time, 4 = most of the time, and 5 = almost always) [44]. The two-item global leadership scale was reported to have Cronbach's alpha of 0.78 [44]. Our research found a good Cronbach's alpha of 0.85, with Cronbach's alpha of 0.83 for physicians and 0.86 for nurses. The total global leadership score is a sum of the two items and ranged from 2 to 10, indicating the extent to which participants perceived themselves as leaders in their clinical practice.

#### 3.2.2. Job-Related Measures

We used a single item to measure job satisfaction and a single item to measure workload. For job satisfaction, physicians and nurses were asked to rate how satisfied they were with their current job in the hospital on a scale from 0 (completely dissatisfied) to 100 (completely satisfied) [45]. For workload, they were asked to rate how much workload they experienced on a scale from 0 (none) to 100 (a lot) [46]. The use of single-item measures is justified under time constraints by research showing that it measures the same as, or is even more inclusive than a sum of items, when multiple items cannot grasp the range of variables that influence the measured construct [45–48].

#### 3.2.3. Physicians as Bridge-Builders

Our study used items to measure attitudes and behaviours that improve team cohesion, to indicate physicians' bridge-building behaviours towards other physicians. The four items are based on a subscale from a questionnaire measuring interprofessional collaborative competency—the Chiba Interprofessional Competency Scale (CICS29)—that evaluates competencies based on behaviour [49]. This subscale was reported to have a Cronbach's alpha of 0.83 [49]. Our research adjusted the items by adding an explicit group towards whom the behaviours were expressed. For example, questioning “I consciously create opportunities for communication with physicians from another specialty,” instead of “I consciously create opportunities for communication with other professionals.” Respondents were asked to indicate to what extent they agreed or disagreed (1 = strongly disagree, 2 = disagree, 3 = neutral, 4 = agree, and 5 = strongly agree) with a statement. We found an acceptable Cronbach's alpha of 0.75 for these items that were administered only to physicians. The total score was an average of the four items and ranged from 1 to 5. Higher scores represented more positive attitudes and behaviours for team cohesion.

#### 3.2.4. Nurses as Bridge-Builders

The questionnaire on the International Organization of an Intensive Care unit is an instrument designed to assess the opinion of healthcare professionals on the organization for which they work [50]. The scale “multidisciplinary relations and communications” captures the relationships among physicians, as well as between physicians and nurses from a nurses' viewpoint. Respondents are asked to indicate to what extent they agreed or disagreed (1 = strongly disagree, 2 = disagree, 3 = neutral, 4 = agree, and 5 = strongly agree) with a statement. Five statements explicitly captured the relationships between nurses and physicians, two statements the information processing by physicians, and two items the relationships between physicians (see Table 1) [50]. To assess nurses' bridge-building capabilities, we decided to delete the two items that measured the relationships between physicians. The seven items together provided Cronbach's alpha of 0.71. The total score is an average of the seven items and ranged from 1 to 5; the higher scores reflect a more positive view on multidisciplinary relations and communication.

### 3.3. Analysis

Statistical analyses were performed with IBM SPSS Statistics version 27. Descriptive statistics were used to analyse professionals' background characteristics (gender, profession, and tenure characteristics), clinical leadership, job satisfaction, workload, and bridge-building behaviours. Independent samples *t*-tests were run to compare physicians and nurses CLS total and factor scores. Cohen's *d* effect size was computed for the differences in total and factor scores. Pearson product-moment correlations were used to describe coherence between variables. The other hypotheses were tested with simple regression analysis. Six simple regression analyses were run with clinical leadership as the independent variable and job satisfaction, workload, or the relationship with (other) physicians as the dependent variable.

### 3.4. Ethical Considerations

The ethics review board decided that our study was outside the scope of the Netherlands' medical research involving human subjects act, especially because the study focused on professionals, instead of patients (METC-LDD-2019-Z19.019). Respondents were informed of the purpose of the research and participation in the survey was entirely voluntary. Participants agreed participating in the survey before answering the questions, and their identities are kept confidential.

## 4. Results

The descriptive characteristics of the study sample are displayed in Table 2. In total, 139 physicians and 439 nurses responded to the survey, an average response rate of 46.5%. Unfortunately, 39 physicians and 110 nurses did not complete all fifteen items of the questionnaire of main interest and were excluded from analyses.

Independent sample *t*-tests were conducted to compare the clinical and global leadership scores between physicians and nurses (see Table 3). Nurses scored slightly higher on clinical leadership (*M* = 4.08, SD = 0.36) than physicians (*M* = 4.00, SD = 0.41), but this difference was not significant (*t* (427) = −1.77, *p* = 0.08, two-tailed). There was a significant difference in the global leadership scores for nurses (*M* = 5.99, SD = 1.87) and physicians (*M* = 6.62, SD = 1.63; *t* (327) = 3.04, *p* = 0.003, two-tailed), in which physicians scored higher. The difference in the means (mean difference = 0.63, 95% CI [0.22, 1.04]) was small (eta squared = 0.03/Cohen's *d* = 0.35).

Coherence between variables (clinical leadership, global leadership, job satisfaction, workload, and bridge building) was investigated using Pearson product-moment correlation coefficients (see Table 4). There was a statistically significant positive association between clinical leadership and global leadership for physicians (*r* = 0.49, *n* = 100, *p* < 0.001) and nurses (*r* = 0.37, *n* = 329, *p* < 0.001). For physicians, their leadership (clinical and global) was significantly associated with job satisfaction, whereas, for nurses, there was no significant association between their leadership and job satisfaction. Interestingly, for both physicians and nurses, their leadership was statistically significant and positively associated with bridge building. Although higher workload was associated with lower job satisfaction for nurses, this was not the case for physicians.

A series of simple regression analyses were used to test if clinical leadership of both physicians and nurses predicted job satisfaction, workload, and bridge building (see Table 5). Clinical leadership did not significantly predict a nurses' job satisfaction (*β* = 0.10, *p* = 0.082) nor workload (*β* = 0.03, *p* = 0.558). Clinical leadership did predict significantly a physicians' job satisfaction (*β* = 0.35, *p* < 0.001) but not their workload (*β* = 0.11, *p* = 0.283). Also, physicians' clinical leaders were more likely to express positive attitudes and behaviours towards physicians from other specialties (*β* = 0.40, *p* < 0.001), while nurse clinical leaders were more likely to rate positively their relation and communication with physicians (*β* = 0.20, *p* < 0.001).

## 5. Discussion

Nurses and physicians are both seen as important driving forces behind initiatives to improve patient-centred care. They are expected to increase alignment and integration between healthcare professionals, especially by taking on informal roles as bridgebuilders. This is part of their role as clinical leaders: “a clinical leader is a healthcare professional who is directly involved in clinical care and continuously puts effort in the improvement of care and inspires and motivates others to do the same.” As physicians and nurses can both perform the clinical leadership role, but clearly have a different position in healthcare, we aimed to understand differences and similarities between physicians' and nurses' clinical leadership behaviours and explored how this relates to their interdisciplinary collaborative behaviours.

In contradiction to our first hypothesis, our results suggest that physicians and nurses show similar clinical leadership behaviours. This is in contrast with literature suggesting that nurses have a more subordinate position that may discourage them to take on such a role [20, 21] and that nurses believe they lack the necessary knowledge and skills to perform a clinical leadership role [22]. However, at the same time, our findings show that physicians are more likely to perceive themselves as leaders in clinical practice than nurses. It may therefore be that nurses are less likely to define the tasks related to clinical leaders, such as bridgebuilding and initiating change, as leadership. Clark argues that nurses equate leadership with authority and specific job titles rather than a way of thinking or behaving [51]. Another, but related explanation could be that nurses use less dominant strategies to fulfil these roles and therefore do not consider it to be leadership [52, 53]. As to our knowledge, research comparing clinical leadership behaviour between nurses and physicians is lacking, we were not able to corroborate these possible explanations. It therefore should be part of future research.

In contrast to hypothesis IIa, nurses who showed clinical leadership behaviours did not perceive a higher workload. We expected nurses to not be facilitated and supported enough to take the informal leadership position [25] and that they would therefore perceive higher workload. However, maybe clinical leadership behaviours (e.g., bridge building and initiating change) are in fact not that remote from what nurses already do and nurses perceive it as part of their profession, not expecting extra compensation. Furthermore, our findings show that increased clinical leadership behaviour did not affect nurses' job satisfaction, while literature suggests that nurses who show clinical leadership and experience professional autonomy will be more satisfied with their jobs [54, 55]. However, as discussed before, nurses are less likely to see themselves as “leaders” and are maybe also not perceived as leaders by others. They may therefore not receive the recognition and autonomy that they equate with formal leadership positions (as suggested by Clark [51]). It may also be that they do not “claim” autonomy and status, so they will not clash with other professionals (and lose their support) but instead use more nonconfrontational strategies to initiate change [52, 53].

In line with hypothesis IIb, our research showed that physicians who express more clinical leadership behaviours reported higher satisfaction with their job, while it had no impact on their perceived workload. We argued based on literature that for physicians, being an informal leader is embedded in their professional identity and so taking on a clinical leadership role does not lead to increased workload but provides an opportunity for professional development, leading to higher job satisfaction [30]. Although this seems a likely explanation for our findings, follow up studies may be relevant to better understand the relationship between clinical leadership behaviour and job satisfaction both for nurses and physicians.

In line with hypothesis III, physician clinical leaders show more positive attitudes and behaviour towards physicians from other specialties. Other studies already suggest that clinical leaders can build bridges with other groups such as managers [32, 34, 35]. Our study adds that bridge building of these clinical leaders also relates to other medical specialities.

In contradiction to hypothesis IV, nurses showing more clinical leadership behaviours rated their communication and relationships with physicians better. Based on the literature, we expected that physicians might resist nurses taking on leadership roles as this affects their existing leadership position [36, 42]. However, as suggested earlier, it might be that nurses use nonconfrontational strategies to perform their role as clinical leaders and are therefore not perceived as a threat to physicians. At the same time, some research studies suggest that complementary leadership between doctors and nurses is quite possible when they share clear goals [39]. The bridge-building capabilities of these clinical leaders might then improve the relationship between them. This could mean that clinical leadership of doctors and nurses can coexist and complement each other.

As healthcare is reforming to a patient-centred approach, modern healthcare leadership needs to align with quality improvements, such as innovation, clinical effectiveness, and patient experience [56–58]. Because nurses as a profession, at least in hospitals, have the most intensive and direct involvement with patients, it seems suitable for them to act as clinical leaders to promote patient-centred care [10, 59]. Nurse clinical leaders are likely to position themselves as subordinate (followers) to physicians to influence how physicians lead, while not threatening their position and status [52, 53]. Although this may be a successful strategy, for example, for building bridges, it may also reduce their impact in making other substantial changes in health care. Improving health care as a clinical leader may sometimes require more dominant strategies to convince and encourage healthcare professionals who are resistant to change [56].

Unfortunately, our research does not provide a conclusive answer to this interesting discussion. We believe that this discussion is valuable as more dominant position of nurses may be required to take advantage of their ability to indicate problems in the patient safety domain and their appreciation of the significance of interprofessional collaboration, compared to physicians [20, 42]. Therefore, we suggest future research to investigate the clinical leadership role of nurses, the strategies they (can) use as a leader to make changes effectively towards more patient-centred care and strategies to deal with resistance. Two questions that based on literature should be addressed in more detail in future research are as follows: how to educate nurses to become clinical leaders [60] and how perceived and actual influence of nurses can be improved [38]. It would also be important to study strategies in which an organization contributes to more equality between physicians and nurses by, for example, formalizing the role of nurses in leadership positions. Another interesting direction for future research might be to investigate how possible conflicts between healthcare professionals can be managed better to gain benefits, instead of negative outcomes.

### 5.1. Limitations

As with other cross-sectional research, certain limitations applied to our study. First, although we believe our proposed causal relationships between the constructs are plausible, they cannot be determined on cross-sectional data only and require further investigation. Second, we used self-reported measures by the same group of respondents that can cause common method and common source bias. However, this risk was reduced by using different scales for predictor and outcome variables. Third, we cannot convincingly say the view of our respondents necessarily represented the view of nonrespondents as we do not have insights in the features of the nonresponse. This is despite our sample representing the diversity in physician and nurse workforce for a hospital with a variety of represented medical specialties, units, experience on the job, and gender. Fourth, although we believe that the CLS fitted our research, based on multiple arguments and an acceptable Cronbach's alpha, it had not been designed for or tested with physicians before. Fifth, although the current study was not focused on impact of COVID, data were gathered during the COVID pandemic. This might have been a catalyst for building bridges as the pandemic showed closer interdisciplinary collaboration supported more efficient management of care capacities, brought a sense of cohesiveness, and increased recognition of various disciplines [61, 62]. Sixth, we did not differentiate in nursing roles while this might have provided additional insight as literature shows that nurses in an organising role (e.g., arrange patient flow and start and steer quality improvement) can act as bridge builders between professionals and management [63–65]. Despite these limitations, we believe that our study provided relevant insight into the similarities and differences between nurses' and physicians' clinical leadership behaviour and contributes to current scientific and practical debates on the changing roles of healthcare professionals.

## 6. Conclusion

Based on their position in hospitals, nurses have the most frequent and direct contact with patients. Therefore, it seems inevitable for nurses to promote patients' perspectives and promote patient-centred care as part of their clinical leadership role. However, nurses less often perceived themselves as clinical leaders compared to physicians, despite showing similar suitable behaviours. The discussion following our results gives reason to presume that nurses from their nondominant position use more nonconfrontational strategies to exert leadership influence. Although this may be a successful strategy as it enables building bridges between nurses and physicians, it may sometimes require more dominant strategies to convince and encourage healthcare professionals to change.

## Figures and Tables

**Table 1:** Items measuring multidisciplinary relations and communications.

Statement	Reverse coded?	Relationship
I find it easy to discuss openly with the unit's physicians	No	Nurse-physician
I have sometimes been poorly informed by the unit's physician	Yes	Nurse-physician
Communication among the unit's physicians is very open	No	Among physicians
I often have to check the accuracy of the information I receive from the unit's physicians	Yes	Nurse-physician
I find it very enjoyable to talk with the unit's physicians	No	Nurse-physician
When physicians talk with each other in this unit, they understand each other well	No	Among physicians
The information exchanged by the unit's physicians is sometimes inaccurate	Yes	Information processing
It's easy to ask for advice from the unit's physicians	No	Nurse-physician
I feel that some of the unit's physicians do not fully understand the information they receive	Yes	Information processing

**Table 2:** Descriptive statistics of background characteristics, leadership, and outcomes.

	Categories	Range of scale	Physicians (*n* = 100)	Nurses (*n* = 329)
			Percent or mean (SD)	Percent or mean (SD)
Gender	Male		(45.0%)	(5.5%)
	Female		(39.0%)	(80.5%)
	Unknown		(16.0%)	(14.0%)

Formal leadership function	Yes		(24.0%)	(2.1%)
	No		(72.0%)	(97.0%)
	Unknown		(4.0%)	(0.9%)

Years working in profession	0–5		(23.0%)	(28.7%)
	5–15		(30.0%)	(27.8%)
	>15		(32.0%)	(31.8%)
	Unknown		(15.0%)	(11.9%)

Years working in hospital	0–5		(29.5%)	(29.8%)
	5–15		(31.3%)	(27.1%)
	>15		(2.0%)	(31.3%)
	Unknown		(14%)	(11.8%)

Clinical leadership		1–5	4.00 (0.41)	4.08 (0.36)

Global leadership		2–10	6.62 (1.63)	5.99 (1.87)

Job satisfaction		1–100	80.69 (13.28)	79.95 (11.92)

Workload		1–100	67.83 (19.70)	68.42 (21.11)

Bridge building		1–5	3.48 (0.47)	3.61 (0.61)

**Table 3:** Mean scores and differences between physicians' and nurses' clinical and global leadership.

Leadership score	Profession^*∗*^	Mean	SD	SEM	*T*	df	*p*value	Partial eta squared/Cohen's d
Clinical leadership (1–5)	Physicians	4.00	0.41	0.04	−1.77	427	0.078	0.01/−0.26
	Nurses	4.08	0.36	0.02				

Global leadership (2–10)	Physicians	6.62	1.63	0.16	3.04	427	0.003	0.03/0.35
	Nurses	5.99	1.87	0.10				

^*∗*^Physicians *n* = 100; nurses *n* = 329.

**Table 4:** Pearson correlation and significance between variables.

	1	2	3	4	5
*Physicians*					
(1) Clinical leadership	1				
(2) Global leadership	0.49^*∗∗∗*^	1			
(3) Job satisfaction	0.35^*∗∗∗*^	0.31^*∗∗*^	1		
(4) Workload	0.11	0.02	−0.03	1	
(5) Bridge building^1^	0.40^*∗∗∗*^	0.31^*∗∗*^	0.14	0.14	1

*Nurses*					
(1) Clinical leadership	1				
(2) Global leadership	0.37^*∗∗∗*^	1			
(3) Job satisfaction	0.10	−0.02	1		
(4) Workload	0.03	0.03	−0.20^*∗∗∗*^	1	
(5) Bridge building^2^	0.20^*∗∗∗*^	0.14^*∗*^	0.28^*∗∗∗*^	−0.10	1

^1^Bridge building measured as attitudes and behaviours that improve team cohesion between physicians. ^2^Bridge building measured as communication and relationships with physicians *p* value significant (two-tailed) at level: ^*∗*^<0.05, ^*∗∗*^<0.01, and ^*∗∗∗*^<0.001.

**Table 5:** Results of regression analyses.

	Physicians		Nurses	
	*β*	*p* value	*β*	*p* value
*Job satisfaction*				
Clinical leadership	0.35	<0.001	0.10	0.082
*R* ^2^ (adjusted)	0.11		0.01	
*F* test	12.41^*∗∗*^		3.05	

*Workload*				
Clinical leadership	0.11	0.283	0.03	0.558
*R* ^2^ (adjusted)	0.00		0.00	
*F* test	1.17		0.34	

*Bridge building* ^*1*^				
Clinical leadership	0.40	<0.001	0.20	<0.001
*R* ^2^ (adjusted)	0.15		0.04	
*F* test	17.26^*∗∗*^		11.92^*∗∗*^	

^*∗*^ *F* test is significant at the 0.05 level (two-tailed). ^*∗∗*^*F* test is significant at the 0.01 level (two-tailed). ^1^For physicians measured as attitudes and behaviours that improve team cohesion between physicians and for nurses measured as communication and relationships with physicians. Clinical leadership predicting job satisfaction, workload, and relationships with (other) physicians.

## Data Availability

The survey data used to support the findings of this study are not publicly available due to privacy reasons but may be released upon reasonable application to the authors, who can be contacted via the corresponding author.

## Supplementary Materials

Supplementary Materials S1: STROBE Statement. Checklist of items that should be included in reports of observational studies.
